# Host–guest-driven color change in water: influence of cyclodextrin on the structure of a copper complex of poly((4-hydroxy-3-(pyridin-3-yldiazenyl)phenethyl)methacrylamide-co-dimethylacrylamide)

**DOI:** 10.3762/bjoc.10.259

**Published:** 2014-10-24

**Authors:** Nils Retzmann, Gero Maatz, Helmut Ritter

**Affiliations:** 1 Institute of Organic Chemistry and Macromolecular Chemistry, Heinrich-Heine-University Duesseldorf, Universitaetsstraße 1, D-40225 Duesseldorf, Germany

**Keywords:** cyclodextrin, copolymer, copper(II) sulfate, supramolecular, water complex

## Abstract

In the present work we report the synthesis of poly((4-hydroxy-3-(pyridin-3-yldiazenyl)phenethyl)methacrylamide-co-dimethylacrylamide) and its reversible optical and complex-forming properties due to copper and cyclodextrin (CD) interactions. Color changing effects are characterized by UV–vis spectroscopy and the supramolecular behavior is investigated by dynamic light scattering experiments.

## Introduction

In recent years, the interest in stimuli-responsive polymers increased exponentially [1]. Many polymers have been described, showing sensitivity towards e.g. light, pH and heat [2–4].

Accordingly, polymer-based fluorescent and colorimetric chemosensors have attracted great attention due to several important advantages such as their simplicity of use, or their versatile structural designs [5].

In this connection, also the interactions of suitable polymers with cyclodextrin (CD) have been studied extensively, causing for example a blue to red color transition of polydiacetylenes (PDAS) due to the formation of inclusion complexes [6–7].

Azo dyes, with their remarkable ability to form stable azo–metal chelate complexes with outstanding thermal and optical properties have been studied widely and led to an increased usage of dyes in the field of optical recording media [8–9].

However, up to now, only little is known about the interactions of azo–metal chelate complexes in supramolecular structures with CD as a modulator for macromolecular effects. Few reports dealt with the reversibility of CD–dye hydrogels by addition of metal ions, as well as the sensing of copper ions using cyclodextrin–dye rotaxane [10–11].

Herewith we thus wish to describe our recent results about the synthesis of a water-soluble polymer bearing a novel azo dye and the presumed formation of complexes with Cu ions and CD in aqueous media under respect of color change.

## Findings

**Scheme 1:**
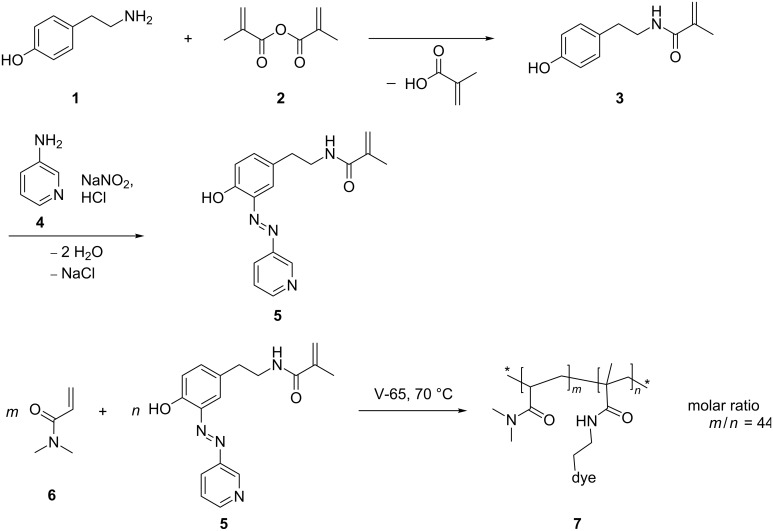
Synthesis of *N*-(4-hydroxy-3-(pyridin-3-yldiazenyl)phenethyl) methacrylamide (**5**) and preparation of its water-soluble copolymer **7**.

The aim of this work was to synthesize the polymerizable azo dye *N*-(4-hydroxy-3-(pyridin-3-yldiazenyl)phenethyl)methacrylamide (**5**) which should potentially be able to form stable azo–metal complexes in aqueous solutions. Therefore tyramine (**1**) was selectively methacrylated at room temperature followed by a diazotation coupling with 3-aminopyridine (**4**) leading to *N*-(4-hydroxy-3-(pyridin-3-yldiazenyl)phenethyl)methacrylamide (**5**). Subsequently, the azo dye containing polymer **7** was synthesized through free radical copolymerization of **5** with *N*,*N*-dimethylacrylamide (**6**) using 2,2’-azobis(2,4-dimethyl)valeronitril (V-65) as an initiator (Scheme 1).

**Figure 1:**
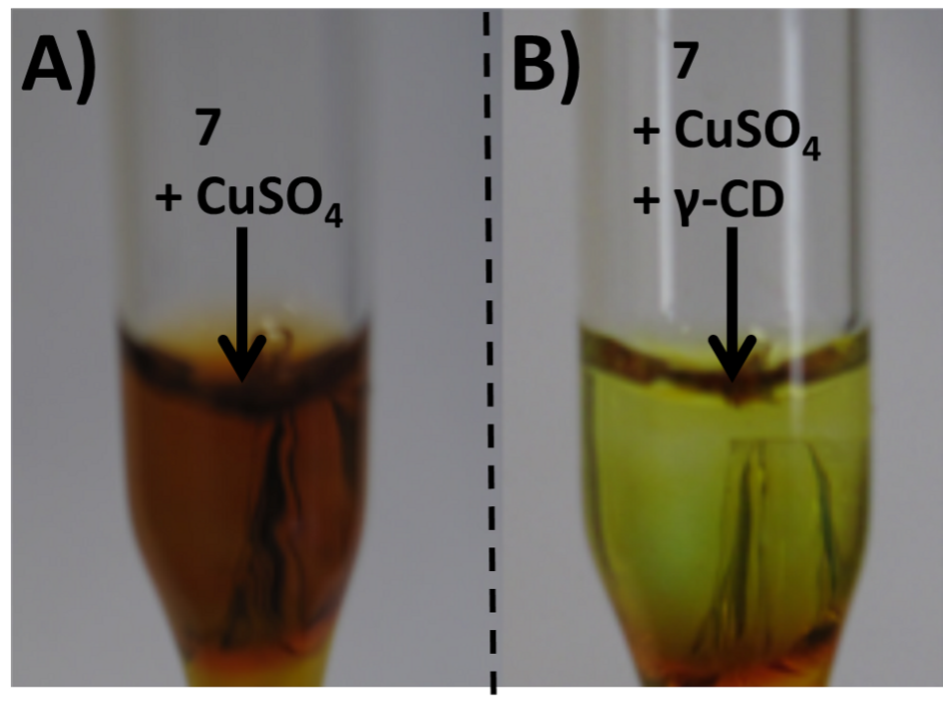
Color-changing effects of polymer **7** upon addition of A) CuSO_4_ and B) CuSO_4_ and γ-CD in a 50:50 vol % in methanol/water solution.

The resulting copolymer **7** is orange colored. The color-changing effects of **7** upon addition of CuSO_4_ alone and in the presence of **γ**-CD were investigated and representative images are shown in Figure 1.

The addition of CuSO_4_ to polymer **7** in a water/methanol mixture caused a color change from orange to red, which could be reversed by subsequent addition of **γ**-CD (Figure 1). Thus, we can anticipate a donor–acceptor-type bonding of the electron-rich azo dye with copper ions, that can be displaced by the addition of **γ**-CD [12].

**Figure 2:**
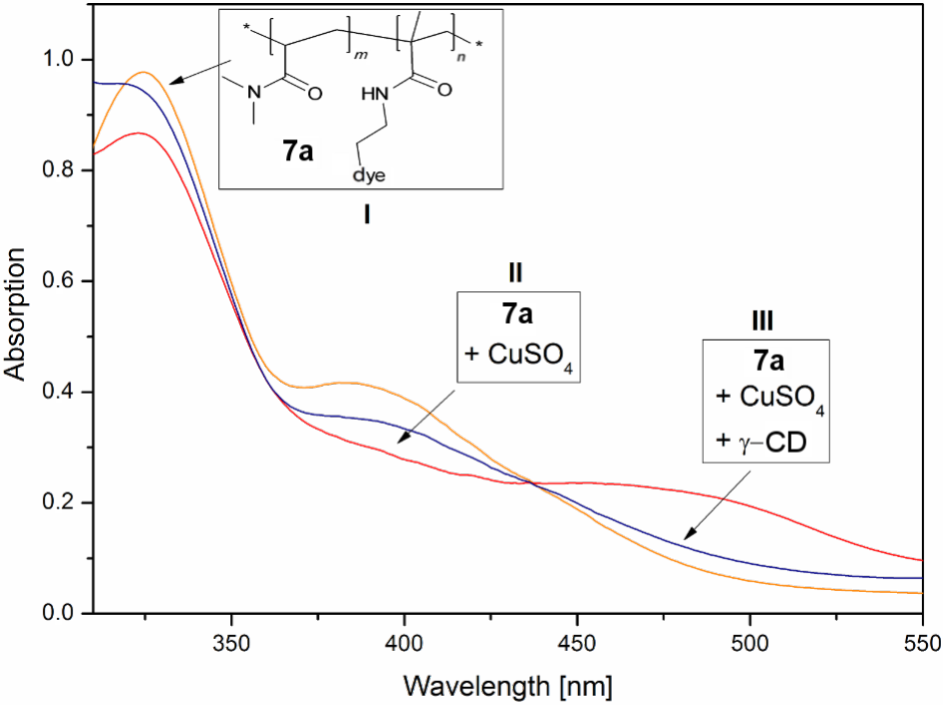
UV–vis absorption spectra of (orange) the solved copolymer **7** with the induced shifts by addition of (red) copper(II) sulfate and (blue) γ-CD in an aqueous methanol medium.

Subsequent UV–vis spectroscopy allowed us to characterize the above mentioned color changes in the diluted state (Figure 2).

Hereby a bathocromic shift of the peak maximum at 380 nm to 475 nm of **7** was observed due to complexation with CuSO_4_ which is reversible after γ-CD addition.

**Figure 3:**
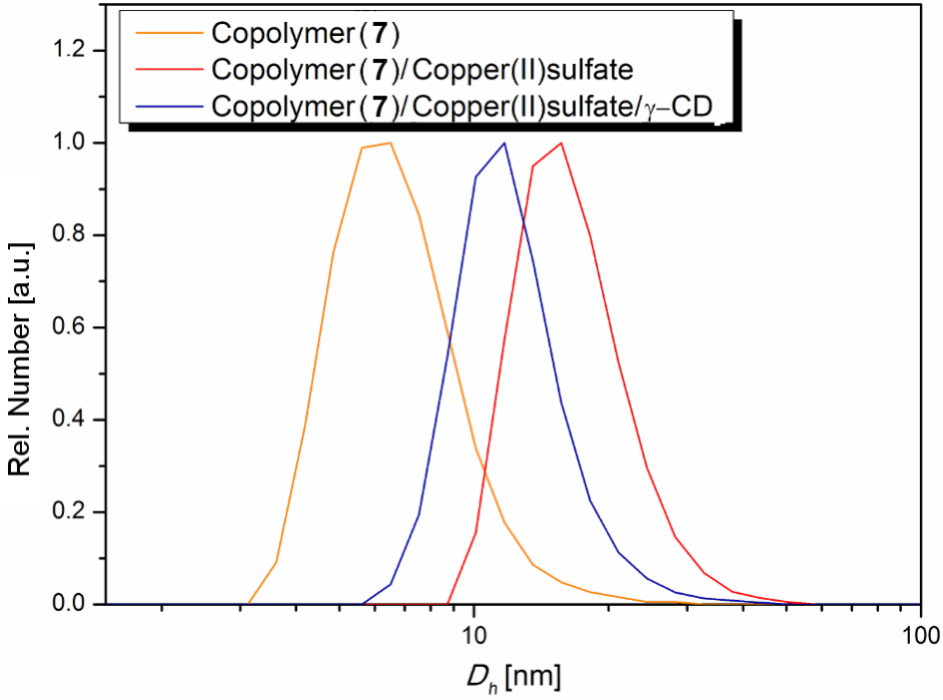
Number average particle size distribution of **7** obtained by DLS experiments.

Assuming an intermolecular aggregating effect due to ionic interactions between the covalently attached azo dyes in **7** and Cu ions in solution, dynamic light scattering (DLS) experiments in a water/methanol solution were carried out (Figure 3). It was found that, by introducing copper ions to **7** the hydrodynamic diameter (D_h_) increased from 11 to 16 nm, while the addition of γ-CD reverses the aggregation effect and reduces the D_h_ correspondingly. This effect, however it is not fully reversible clearly shows the potential ability of **7** to form high molecular aggregates caused by reversible azo-copper complexes in aqueous media.

In result we could show that supramolecular structures and metal complexes open up a wide field for the development of novel stimuli-responsive polymer materials. We thus described that the simple addition of copper and γ-CD to a poly((4-hydroxy-3-(pyridin-3-yldiazenyl)phenethyl)methacrylamide-co-dimethylacrylamide) copolymer leads to reversible color changes and further to aggregation processes in the diluted state. The described phenomena are easy to observe with the naked eye.

## Supporting Information

File 1 Experimental.
