# Supplementary material for: Host–guest-driven color change in water: influence of cyclodextrin on the structure of a copper complex of poly((4-hydroxy-3-(pyridin-3-yldiazenyl)phenethyl)methacrylamide-co-dimethylacrylamide)
Source: Beilstein J Org Chem. 2014 Oct 24;10:2480–3. doi: 10.3762/bjoc.10.259 (PMC4222395; doi:10.3762/bjoc.10.259)
Supplement: File 1 — Experimental. [file Beilstein_J_Org_Chem-10-2480-s001.pdf]

Supporting Information
for
Host–guest-driven color change in water: influence of cyclodextrin on the structure of a copper complex of poly((4-hydroxy-3-(pyridin-3-yldiazenyl)phenethyl)methacrylamide-co-dimethylacrylamide)

Nils Retzmann, Gero Maatz and Helmut Ritter*

Address: Institute of Organic Chemistry and Macromolecular Chemistry, Heinrich-Heine-University Duesseldorf, Universitaetsstraße 1, D-40225 Duesseldorf, Germany

Email: Helmut Ritter - h.ritter@uni-duesseldorf.de

*Corresponding author

Experimental

General

All reagents and solvents were commercially available (Sigma-Aldrich) and used without further purification. γ-CD purchased from Wacker Chemie GmbH, Burghausen, Germany was used after drying in an exsicator over P_4O_{10} for 24 hours.

^{1}H-NMR spectras were recorded on a Bruker Avance DRX 300 at 20 °C using MeOD or Deuteriumoxide (99.9%) as solvents.

The absorption spectra were measured in 1 cm quartz cells on a Specord 210 Plus UV–visible spectrophotometer (Analytik Jena AG, Germany) in a 50-50 Vol%.water-methanol solution. For the observed effects copper sulfate was used in shortage and γ-CD in excess.

Dynamic light scattering experiments were carried out with a Malvern Zetasizer Nano, ZS ZEN 3600 at 20 °C. The particle size distribution is derived from a deconvolution of a measured intensity autocorrelation function of the sample by a general purpose method, i.e. the non-negative least squares algorithm, included in the DTS software. For the observed effects copper sulfate was used in shortage and γ-CD in excess.

Synthesis of *N*-(4-hydroxyphenethyl)methacrylamide) (**3**) [1]

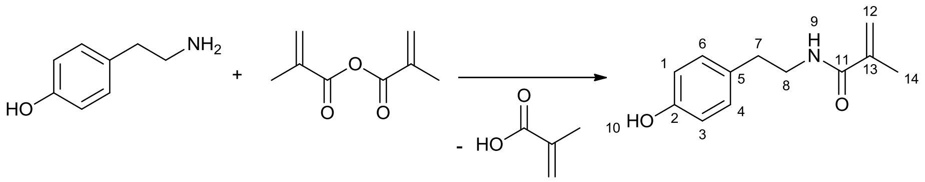

To 0.53 g (3.86 mmol) tyramine solved in 50 ml of methanol, 0.61 ml (4.13 mmol) of methacrylic anhydrid (**2**) were added drop wise. The reaction mixture was stirred at room temperature for 2 hours. Subsequently 100 ml of aqueous sodium chloride solution (10%) and 2 g of solid sodium chloride were added and the mixture was further stirred for 30 minutes before excess salt was being removed by filtration. From the obtained mixture a colorless solid could be isolated after cooling for 2 hours in an ice bath and recrystallization in chloroform. (Yield 59%, Lit. 84,5% [1])

^{1}H-NMR (300 MHz, DMSO-d_6) δ 9.17(s,1H,H.10), 7.94(t,1H,H.9), 7.01-6.95(m, 2H,H.4,6), 6.70-6.64(m,2H,H.1,3), 5.62,5.31(m,2H,H.12), 3.30-3.20(m,2H,H.8), 2.66-2.59(m,2H,H.7), 1.83(s,3H,H.14) [ppm]

GC-EIMS, m/z (M+): 205

MP: 124, Lit. 123°C [1]

Synthesis of *N*-(4-hydroxy-3-(pyridin-3-yldiazenyl)phenethyl)methacrylamide (**5**)

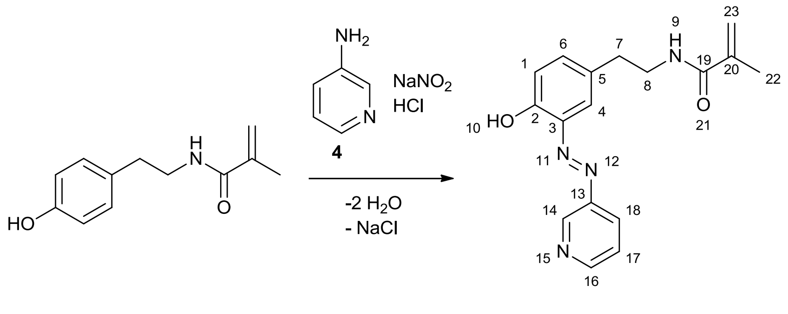

3 5

1000 ml of water were treated with 6 N hydrochloric acid until a pH of 2 was reached. To the acidic solution 0.5 g (5.3 mmol) 3-aminopyridin was added and stirred for 10 minutes at 0 °C. Under heavy stirring 0.44 g (6.3 mmol) sodium nitrite, solved in 50 ml of water, were added. To the cold mixture 1.31 g (6.3 mmol) (**3**) solved in 40 ml alkaline water was added drop wise, while the pH of the reaction mixture was kept below 2. The solution was stirred for 45 minutes below 5 °C, before the pH was adjusted by 2.5 N sodium hydroxide solution to pH 8.5. A dark red precipitate could be isolated after slowly warming to room temperature, readjusting the pH to 6 by 6 N hydrochloric acid, washing with ice-water and air drying on a filter. The obtained solid was further purified by column chromatographic with *n*-hexane and acetone as a solvent. (Yield 38%)

^{1}H NMR (300 MHz, DMSO-d_6) δ 10.66(s,1H,H.10), 9.24(d,1H,H.14), 8.77(dd,1H,H.16), 8.40(m,1H,H.18), 8.05(t,1H,H.9), 7.68(m,1H,H.17), 7.62 (d,1H,H.4), 7.36(dd,1H,H.6), 7.08(d,1H,H.1), 5.66(m,1H,H.23), 5.35(m,1H,H.23), 3.39-3.36(m,2H,H.8), 2.80(t,2H,H.7), 1.91–1.87(m,3H,H.22) [ppm]

FT-IR (diamond): ν(cm^{-1}): 3012(w,-C=C-H_2), 2955- 2925(m, -C-H), 1653(s, Amide) [cm^{-1}]

GC-EIMS, m/z (M+): 310

Synthesis of **7**

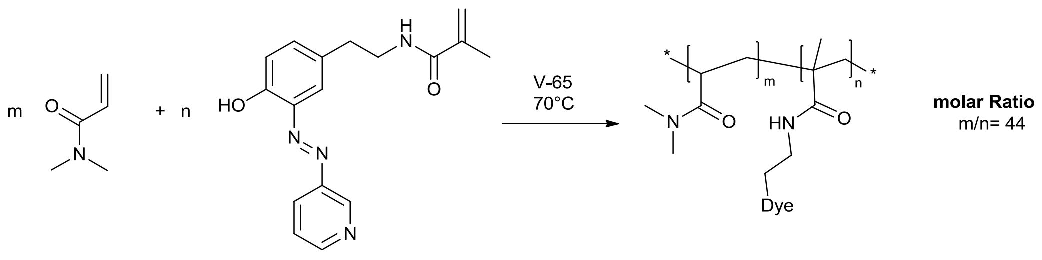

A mixture of 0.5 ml N,N dimethylacrylamide, 35 mg of **5** and 0.5 mg V65 was polymerized in a glass flask at 70 °C for 14 hours. (Yield quantitative)

^{1}H NMR (300 MHz, D_2O) δ 9.02, 8.60, 8.24, 7.75, 7.61, 7.38, 7.00 (Azo, Ar-H), 3.00, 2.77(br,$N(CH_3)_2$), 1.71, 1.24 (br,backbone) [ppm].

References

[1] Jinkerson, D.; Polymerisierbare gelbe Farbstoffe sowie ihre Verwendung in ophthalmischen Linsen, Deutsches Patent 694 16 293 T2, January 27,1999.
